# Duodenal heterotopic pancreatic tissue: a case report and literature review

**DOI:** 10.1093/gastro/gou049

**Published:** 2014-07-25

**Authors:** Rohit Mehra, Aswini K. Pujahari, Shyam S. Jaiswal

**Affiliations:** Department of Surgery, Armed Forces Medical College, Wanowrie, India

**Keywords:** heterotopic pancreas, malignant transformation, small bowel obstruction

## Abstract

Heterotopic pancreas is an aberration in the developmental profile of the pancreas. This entity is rarely symptomatic and its malignant transformation is even rarer. However, when present, it poses a diagnostic dilemma to clinicians, as little help comes from gastroenteroscopy and imaging. Surgical exploration remains the only option at times, and it is the histopathological examination that finally clears the mist. This case report reveals the elusive nature of malignancy in heterotopic pancreas in the duodenum.

## INTRODUCTION

Heterotopic pancreas (HP) is the presence of pancreatic tissue outside the normal anatomical boundaries, with no vascular connection with the main pancreas. Several theories have tried to define this aberration from normal embryological development, but none stands up to scrutiny [1–4]. Though mostly asymptomatic, this condition can have varied presentations and a malignant transformation is extremely rare [5].

## CASE PRESENTATION

A 51-year-old male, with no known comorbidities, presented to us with history of nausea, pain abdomen and post-prandial vomiting for previous three months. Initially he used to have 2–3 episodes of post-prandial vomiting, which progressively increased to 8–10 episodes over a period of three months. He experienced a weight loss of 19 kilograms in the same time. There was no history of distension of abdomen, fever, haematemesis or melena. The physical examination was within normal limits and he was haemodynamically stable. Haematological and biochemical reports were normal.

**Figure 1:**
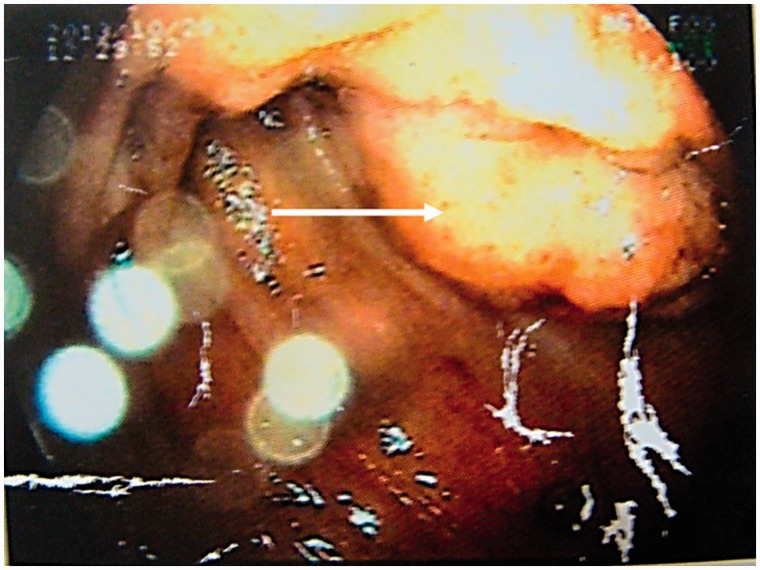
An upper gastrointestinal endoscopy revealed a fleshy mucosal growth (as indicated by the arrow) at the medial wall of the distal third part of the duodenum.

An upper gastrointestinal endoscopy revealed oesophagitis, with a dilated stomach and first and second parts of the duodenum, and a fleshy, mucosal growth in the medial wall of the distal end of the third part of duodenum (Figure 1). The endoscope was able to negotiate beyond the growth. A contrast-enhanced computed tomography scan of the abdomen corroborated the findings of a circumferential growth at the medial wall of the third part of the duodenum, with marked desmoplasia (Figure 2). The biopsies were non-diagnostic. Due to failure of conservative management and a diagnostic dilemma, a surgical option was contemplated.

**Figure 2a:**
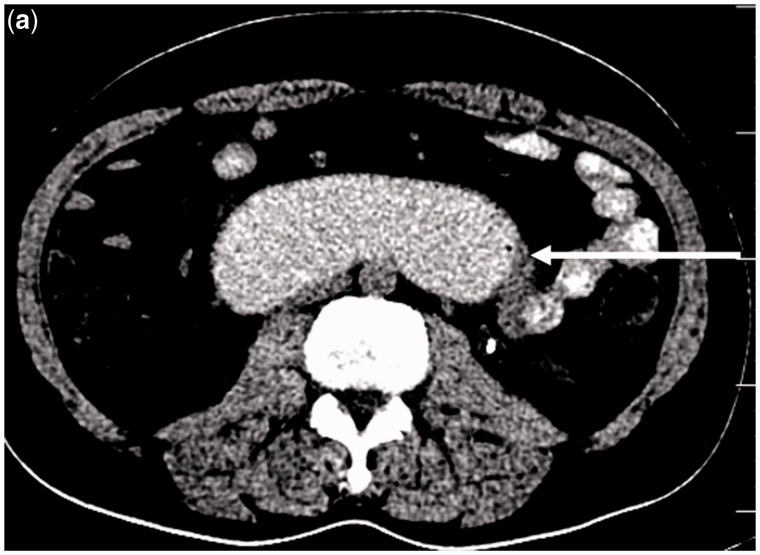
Abdomen CT scan (transverse plane): the area indicated by the arrow shows a circumferential growth seen at the region of the duodeno-jejunal flexure, also involving the medial wall of the fourth part of the duodenum. It seems to extend beyond the serosa into the adjacent mesentery.

**Figure 2b:**
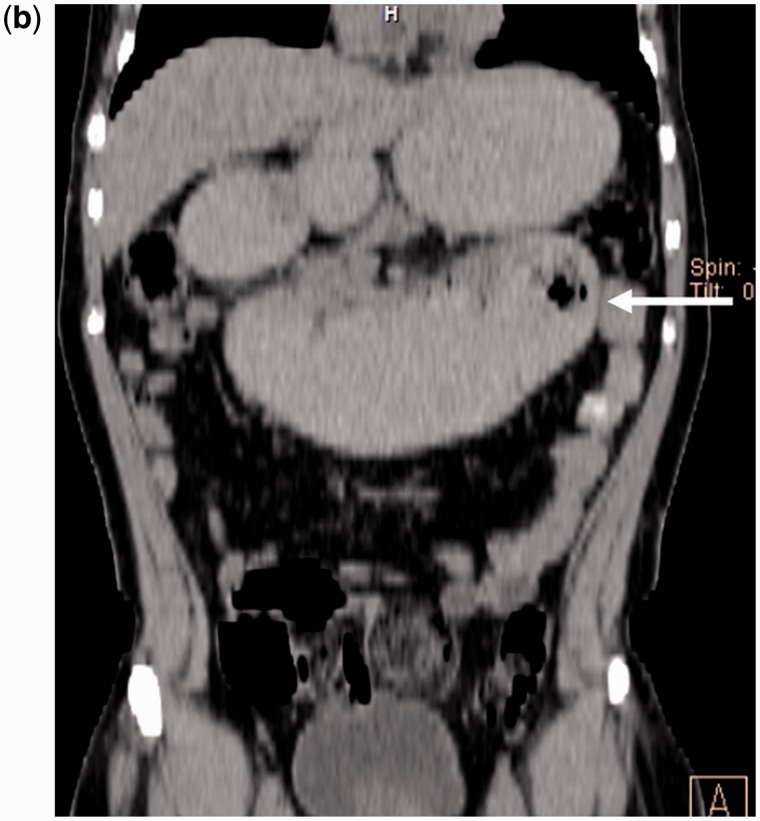
Abdomen CT scan (coronal plane): the area indicated by the arrow showed grossly dilated D1, D2 and D3 segments of the duodenum, with constriction seen at the duodeno-jejunal junction. The small bowel loops are partially collapsed after the lesion.

**Figure 3:**
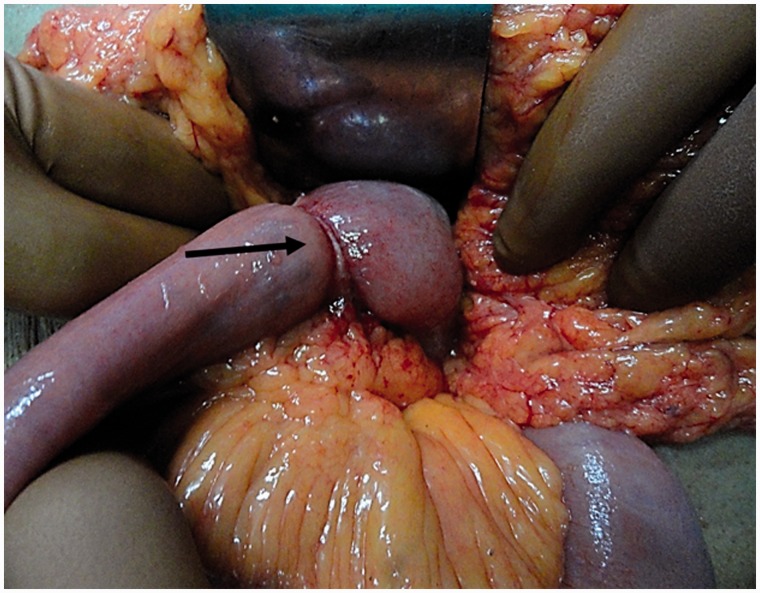
Intraoperative photograph: the arrow points at the lesion involving the duodenum.

**Figure 4:**
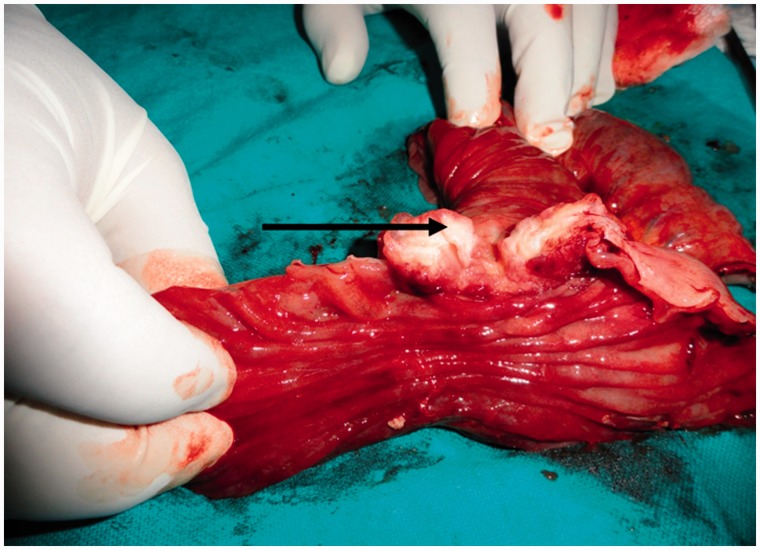
The cut open surface of the post-operative specimen. The arrow shows the lesion involving the duodenum nearly circumferentially.

**Figure 5:**
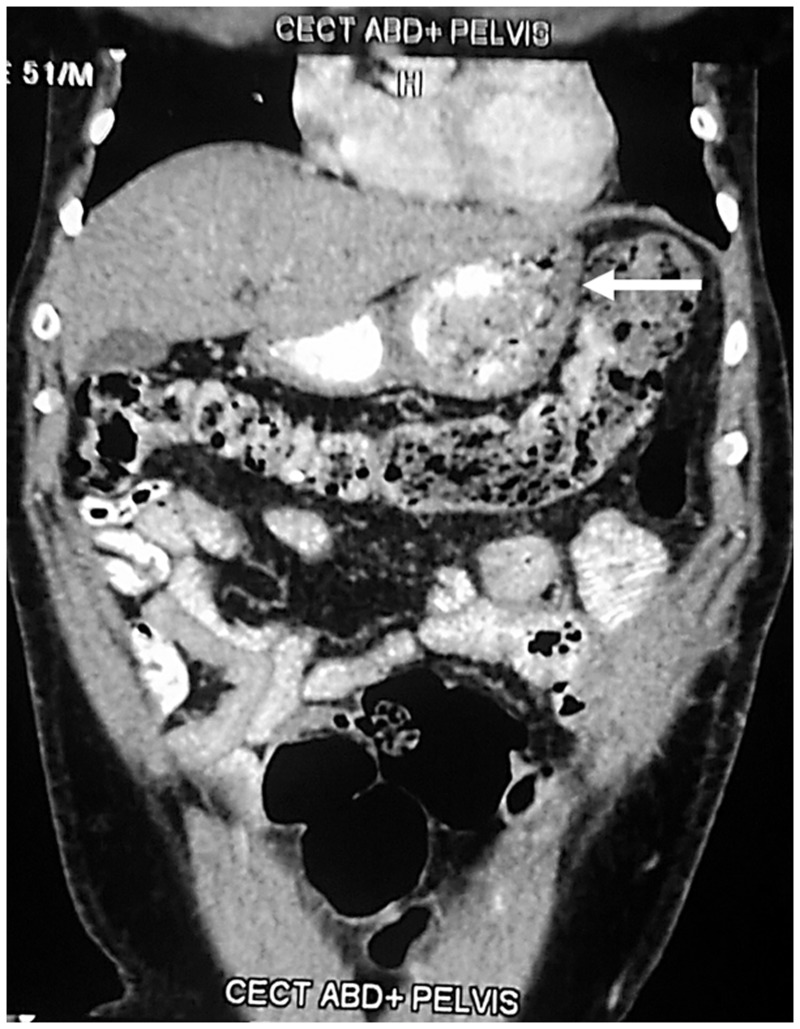
Abdomen and pelvic CT scan at 6 months follow-up reveals no residual disease or recurrence.

Through a mid-line laparotomy, the duodenal lesion was subjected to a segmental resection and end-to-side duodenojejunostomy with a distal feeding jejunostomy. The resected lesion measured 5 × 4 × 3.5 cm and involved the fourth part of the duodenum. A 2 × 1 cm suspected malignant nodule was also resected from the jejunal mesentry, approximately 3 cm from the duodeno-jejunal flexure (Figures 3 and 4). Histopathological analysis of the resected lesion showed the presence of acinar and ductular lobules of pancreas with well-differentiated adenocarcinoma (Heinrich Type II). All the resected margins were negative and there was one lymph node positive out of the six resected with the specimen. The post-operative period was uneventful. There is no evidence of residual disease or recurrence, clinically or on imaging (Figure 5).

## DISCUSSION

The credit for first description of HP goes to Jean Schultz in 1727 [6]. However, the first histological proof came from Klob in 1859 [7]. The world literature reports a widely variable presence of HP in 0.6–13% of autopsies [8, 9]. It has a slight bias towards males and can be found throughout the gastrointestinal tract, the most frequent sites being stomach, duodenum and jejunum [10]. Isolated reports of HP at the gall bladder have also been cited [3].

Although its cause is unclear, three theories have been put forward. The ‘theory of metaplasia’ states that it is the metaplasia in the pancreatic endodermal tissue, during embryological development, that leads to HP [1], while the ‘theory of misplacement’ supposes that, during the period of rotation of the gut, there is misplacement of pancreatic tissue due to fragmentation—before the fusion of the dorsal and ventral anlage—leading to HP later in life [2]. The latest addition is the ‘theory of abnormalities of notch signalling’. This is a type of cell interaction mechanism; in mice, it has been proven that abnormal expression of pancreas-specific transcription factor 1a (PTF1A) leads to formation of HP [3, 4].

HP is usually an asymptomatic aberration but, as and when clinical symptoms arise, the manifestations are site-specific. The usual symptoms are nausea, vomiting and abdominal epigastric pain, increasing in severity in the post-prandial period. Malignant transformation of the HP is exceedingly rare. All disease conditions that can affect the normal pancreatic tissue, such as pancreatitis and pseudocyst formation, can occur in HP. Very few cases of duodenal adenocarcinoma arising from HP have been reported in the world literature [11, 12]. Owing to the varied presentations, the diagnosis of HP is difficult. No specific diagnostic methods can be employed and a pre-operative diagnosis is seldom possible [13]. Few reports suggest that endoscopic ultrasound and computed tomography may help in distinguishing HP from other submucosal gastrointestinal tumours, but no long-term studies are there to support the claim [14, 15].

Confirmatory diagnosis is usually histological. Histologically, HP has been classified into three types by Heinrich in 1909 [16]. Type I (the commonest variety) shows the presence of ducts, acini and islets of Langerhans cells; Type II shows only ducts and acini, while Type III has only ducts. The definition was later modified by Gasper *et al.* Type I had ducts, acini and Langerhans cells, similar to normal pancreas. Type II had ducts only and Type III showed acini and type IV islets [17].

With regard to management, a surgical intervention is essential in a symptomatic patient. Although open surgery was once preferred, the recent world literature boasts several reports of successful laparoscopic and endoscopic interventions [18]. However, medical opinion is divided over the management of an incidental HP. Our patient is currently responding well to gemcitabine- and oxaliplatin-based adjuvant therapy for the pancreas and has responded well to treatment; however, the prognosis for adenocarcinoma in HP is not well known. Future detailed studies might be able to clarify this aspect.

In conclusion, we have described a case of adenocarcinoma arising from HP in the duodenum. This phenomenon is extremely rare and difficult to diagnose pre-operatively. The clinical profile of the patient included nausea, post-prandial vomiting and significant weight loss. The endoscopic findings were inconclusive, as were the biopsies. The diagnosis was established through histopathology after surgery. The rarity of this condition makes the diagnosis and treatment a herculean challenge. We require more detailed and in-depth studies on this matter to solve the elusive riddle of HP.

**Conflict of interest:** none declared.
